# Morphofunctional Organization of the Connections From the Medial and Intermediate Parts of the Central Nucleus of the Amygdala Into Distinct Divisions of the Lateral Hypothalamic Area in the Rat

**DOI:** 10.3389/fneur.2018.00688

**Published:** 2018-08-24

**Authors:** Marie Barbier, Dominique Fellmann, Pierre-Yves Risold

**Affiliations:** Laboratoire de Neurosciences Intégratives et Cliniques, EA481, UFR Sciences Médicales et Pharmaceutiques, Université de Bourgogne Franche-Comté, Besançon, France

**Keywords:** lateral hypothalamus, amygdala, melanin-concentrating hormone, hypocretin, tract tracing

## Abstract

Projections from the central nucleus of the amygdala (CEA) into the lateral hypothalamic area (LHA) show a very complex pattern. After injection of an anterograde tracer (*Phaseolus vulgaris* leucoagglutinin—PHAL) into the medial and intermediate parts of the CEA, we observed that labeled axons converged onto the caudal lateral LHA but provided distinct patterns in rostral tuberal regions. These projections were compared to that of neurons containing the peptides “melanin-concentrating hormone” (MCH) or hypocretin (Hcrt). Because the distribution of these neurons is stereotyped, it was possible to characterize distinct divisions into the LHA. Some of them in the rostral tuberal LHA [the dorsal (LHAd) and suprafornical regions (LHAs)] received a distinct innervation by projections that originated from neurons in respectively anterior or posterior regions of the medial part (CEAm) or from the intermediate part (CEAi) of the central nucleus. Therefore, this work illustrates that projections from the CEAm and CEAi converge into the caudal lateral LHA but diverge into the rostral tuberal LHA.

## Introduction

The lateral hypothalamic area (LHA) receives inputs from a very large number of brain sites and is involved in a large range of functions from ingestive behaviors to the control of behavioral state and sleep/wake cycle (1–6). Many studies have focused on the role of specific neuron populations of the LHA, including the melanin-concentrating hormone (MCH) and hypocretin (Hcrt) containing cells that form conspicuous populations in the tuberal hypothalamus (7–10). However, despite these efforts, we must acknowledge that the internal organization of the LHA is still poorly understood.

One of the main function associated with the LHA is the initiation of feeding behavior (1, 10). Literature data indicates that neuropeptide Y (NPY) and proopiomelanocortin (POMC) neurons from the arcuate nucleus provide metabolic information to second order LHA neurons (MCH, Hcrt) that may then initiate the feeding response (2, 9, 11–16). Other works point to projections from the bed nucleus of the stria terminalis or accumbens nucleus that are important for this response from the LHA, but involving non-MCH and non-Hcrt, glutamatergic, or GABAergic cells (17–19). The central nucleus of the amygdala (CEA) is another telencephalic structure innervating the LHA and is involved in the initiation of feeding (20–26). This nucleus is closely connected to the parasubthalamic nucleus (PSTN), a caudal LHA nucleus devoid of MCH and Hcrt neurons (7, 21). However, the CEA also projects into the perifornical LHA. In a recent study (27), we identified that these projections originate from the medial part of the CEA (CEAm), but also from the intermediate part (CEAi), while the capsular and lateral parts of the CEA send only very sparse projections into the hypothalamus. We noted that mostly the anterior perifornical region of the LHA was innervated by the CEAm/i, but this term is quite vague and this region can be sub-divided as in the Rat Brain Maps (28).

Different strategies can be implemented to increase our knowledge of the LHA organization. One of the most accessible mean to gather meaningful information, is to carefully analyze the distribution pattern of afferents from specific brain sites and compare these patterns with what is known of the cyto- and chemoarchitecture of the LHA.

In the present study, we aimed at carefully analyze the distribution of projections from the CEA into the LHA and compare these distributions with that of MCH and Hcrt neurons. We would then more easily characterize the distribution of the projections from the CEA, with regard to the parceling scheme of this region proposed by Swanson (28).

## Materials and methods

### Animals

All animal use and care protocols were in accordance with institutional guidelines and with the Directive 2010/63/EU of the European Parliament and of the Council of 22 September 2010 on the protection of animals used for scientific purposes. The protocols were approved by Franche-Comté University's Animal Care Committee (protocol number: 2015-002) and the investigators authorized. Four Sprague–Dawley male rats, weighing 300–350 g, were obtained from Janvier (Le Genest-Saint-Isle, France). Rats were housed with a standard 12 h light/dark cycle at a constant room temperature and had free access to the standard laboratory diet and water.

### Tracer injections

PHAL experiments were already reported in previous works (AMY1 in 22, PHAL#1, PHAL#2, PAHL#3 in 27). In two experiments (PHAL#1, PHAL#2) a fluorogold (FG) injection was performed into the PSTN as well as a PHAL injection respectively in the CEAm or the CEAi. Experimental procedures are briefly described below.

Rats were anesthetized with an intraperitoneal (i.p.) injection of a mixture of xylazine and ketamine (1 mg/100 g and 10 mg/100 g of body weight, respectively; Vetoquinol®, France), and placed in a stereotaxic device.

Rats received a unilateral iontophoretic injection of 2.5% PHAL diluted in sodium phosphate buffer saline (NaPBS) pH 7.2. Glass micropipettes (tip diameter: 10–20 μm) were used to inject the PHAL iontophoretically (intermittent current of 5 μA and 7 s on/off time for 20 min). Coordinates were taken from Bregma using the Paxinos' atlas (29). For the CEA, the coordinates were: PHAL#1: AP: −1.53 mm, ML: 3.4 mm, and DV: −8.1 mm; PHAL#2: AP: −1.78 mm, ML: 3.72 mm, and DV: −8.5 mm; and PHAL#3: AP: −2.45 mm, ML: 4.0 mm, and DV: −8.6 mm. Coordinates of AMY 1 were close to those of PHAL#1 but ended more caudally. For the PSTN, the coordinates were AP: −4.2 mm, ML: 1.5 mm, DV: −8.4 mm. To avoid PHAL or FG diffusion along the micropipette track, the micropipette was left in place for another 5 min before being removed.

### Tissue preparation

Rats were deeply anesthetized with an i.p. injection of Pentobarbital (CEVA®, 50 mg/kg). Animals were perfused transcardially with 0.9% NaCl, followed by ice-cold 4% paraformaldehyde (PFA, Roth®) fixative in 0.1 M phosphate buffer (PB) at pH 7.4. Brains were extracted, postfixed for 20 h in the same fixative at 4°C, and cryoprotected by saturation in a 15% sucrose solution (Sigma®) in 0.1 M PB for 24 h at 4°C. Tissues were cut in four series of coronal sections at 30 μm thick, collected in a cryoprotective solution [1:1:2 glycerol / ethylene glycol / phosphate buffer saline (PBS)], and stored at −40°C.

### Enzymatic immunohistochemistry

After rinsing in PBS with 0.3% Triton X100 (PBS-T), free-floating sections were incubated with the anti-PHAL (Rabbit polyclonal, 1:1000, Vector Laboratories®, RRID:AB_2315142) in a solution containing 10% of lactoproteins (commercial dry milk) during 48 h at 4°C. Sections were incubated for 4 h at room temperature in a solution of biotinylated goat anti-rabbit IgG (Vector Laboratories®, RRID:AB_2313606) at a dilution of 1:1000 in PBS-T. Then, sections were placed in the mixed avidin-biotin horseradish peroxidase (HRP) complex solution (ABC Elite Kit, Vector Laboratories®) for 1 h at room temperature. The peroxidase complex was visualized by a 6 min exposure to a chromogen solution containing 0.04% 3,3'diaminobenzidine tetrahydrochloride (DAB, Sigma®) with 0.006% hydrogen peroxide (Sigma®) in PBS. The reaction was stopped by extensive washing in PBS. Sections were mounted on gelatin-coated slides, and then stained in a solution of 1% toluidine blue (Roth®) in water to serve as a reference for cytoarchitectonic purposes. Finally, sections were dehydrated and coverslipped with Canada balsam (Roth®).

### Immunofluorescent staining

After rinsing in PBS-T, free-floating sections were incubated with primary antibodies [anti-Hcrt (Mouse monoclonal, 1:1,000, ANGIO-PROTEOMIE®); anti-MCH [Rabbit polyclonal, 1:1,000, our laboratory (21), RRID:AB_2616562]; anti-PHAL (Goat polyclonal, 1:1,000, Vector Laboratories®, RRID:AB_10000080)] dissolved in PBS-T, 1% bovine serum albumin, 10% lactoproteins, and 0.01% sodium azide or only in PBS-T for 24 h at 4°C. Tissues were washed three times with PBS-T (5 min each) and incubated for 2 h with appropriate secondary antibodies (Cyanine 5, donkey anti-mouse IgG, 1:1,000, Jackson Immunoresearch®, RRID:AB_2340819; Alexa Fluor 488, donkey anti-rabbit IgG 1:1,000, Invitrogen®, RRID:AB_2535792; Cyanine 3, donkey anti-goat IgG, 1:1,000, Jackson Immunoresearch®, RRID:AB_2340411) diluted in PBS-T at room temperature. For triple labeling, this procedure was repeated twice with primaries raised in different species. In some cases, only the FG labeling was detected by epifluorescence under UV illumination. Finally, sections were washed with PBS-T, mounted on gelatin-coated slides and coverslipped with 60/40 glycerol: PBS-T. An adjacent series was always stained in a solution of 1% toluidine blue (Roth) in water to serve as a reference series for cytoarchitectonic purposes.

### Image acquisition and processing

Sections were analyzed on an ApoTome.2 microscope (Axio Imager Zeiss) and images were obtained through a digital camera (Digital Camera Hamamatsu C11440) using the Imager.Z2 software (Zen 2) (ZEN Digital Imaging for Light Microscopy, RRID:SCR_013672). The labeling was observed with appropriate filters: 38 HE Green Fluorescent Protein (BP excitation 450–490, emission 500–550), 43 HE DsRed (BP excitation 538–562, emission 570–540), and 50 Cy5 (BP excitation 625–655, emission 665–715), 49 DAPI (BP excitation 372–401, emission 421–456).

Some pictures were taken using the advanced features “Z-Stack” and “Deconvolution” of the Zen software. Finally, an orthogonal view was performed to obtain a 2D image. Neither additional treatment was made, except the fluorescence intensity. Nomenclature and nuclear parceling are from Swanson (28).

To perform the mapping of PHAL axons in the LHA, photomicrographs of the DAB stained sections and adjacent Nissl stained sections were taken. Drawings of the histological features and axons were made on tracing paper. Drawings were then scanned and transferred as jpeg files into Photoshop. This procedure was chosen as it allowed the most accurate representation of the labeled axon distributions.

## Results

Our initial objective was to compare the distribution of PHAL axons from injection sites in the CEAm and CEAi to the cytoarchitectonic divisions of the LHA as in Swanson (28). Unfortunately, we found very difficult to reliably identify the borders of these divisions on our Nissl stained material and, therefore, we choose to describe these distributions in two steps: (i) a detailed mapping on drawings made from the histological material on which main cytoarchitectonic borders (excluding intra-LHA borders) and fiber tracts are identified; (ii) in a second step, these distributions were compared with that of MCH and Hcrt neurons. The distributions of these neurons are truly stereotyped in the rat hypothalamus and they can be very useful to identify borders of LHA subdivisions as in Swanson et al. (7), or Swanson (28).

### Injection sites

All injection sites reported in this work are from experiments that were already used in previous articles. In the course of our study, we obtained many injections within the borders of the CEA. Only experiments with injection sites restricted to the CEAm/CEAi are reported here as only these parts of the CEA project into the LHA (22, 27). The CEAl and the CEAc do not send significant projections into the LHA (22, 27).

**Figure 1:**
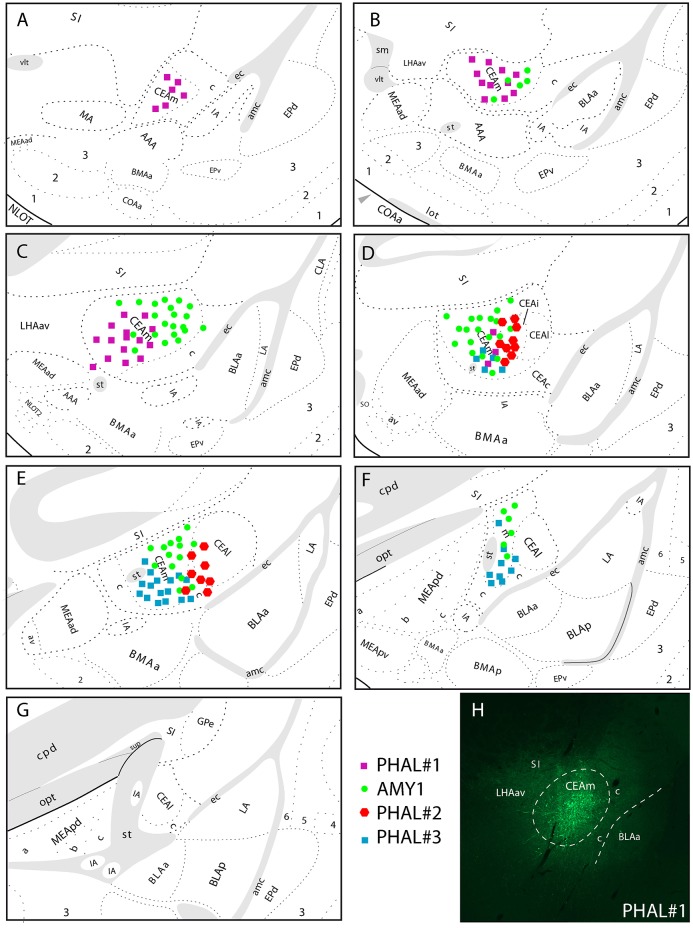
**(A–G)** Line drawings to illustrate the relative extend of PHAL injection sites in the CEAm and CEAi that contributed to significant innervations of the LHA. PHAL#1 was centered in the rostral CEAm **(A–C)**. AMY1 mainly extended into central and caudal CEAm **(D–F)**. PHAL#2 was in the CEAi **(D,E)**. PHAL#3 is restricted to the caudal CEAm. **(H)** Photomicrograph to illustrate the PHAL injection site in the experiment PHAL#1.

The experiment AMY1 was described in Barbier et al. (22). It consisted in a large injection involving most of the CEAm (Figure 1). This injection site extends through the rostrocaudal extend of the CEAm, but with a caudal predominance. It involves a few cell bodies in the CEAi.

The experiment PHAL#1 is a rostral injection extending from the level 24–26 of the Swanson's Brain Maps. It does not involve contamination of the CEAi.

The experiment PHAL#3 is a caudal injection, immediately adjacent to the CEAi, and extends caudally and ventrally into the CEAm with little contamination of the CEAi.

The experiment PHAL#2 is centered in the CEAi with contamination of the CEAm. This injection site overlaps slightly with the PHAL#3 experiment.

In experiments PHAL#1 and PHAL#2, the PHAL injections in the CEA were combined with FG injections in the PSTN. Injection sites in the PSTN are illustrated in the **Figure 4**. The reason for these co-injections is that the PSTN is the major target for CEA projections in the LHA. However, the LHA may as well innervate the PSTN and we wanted to start investigating on some putative intra-LHA microcircuits under CEA control.

### Patterns of innervation of the LHA

**Figure 2:**
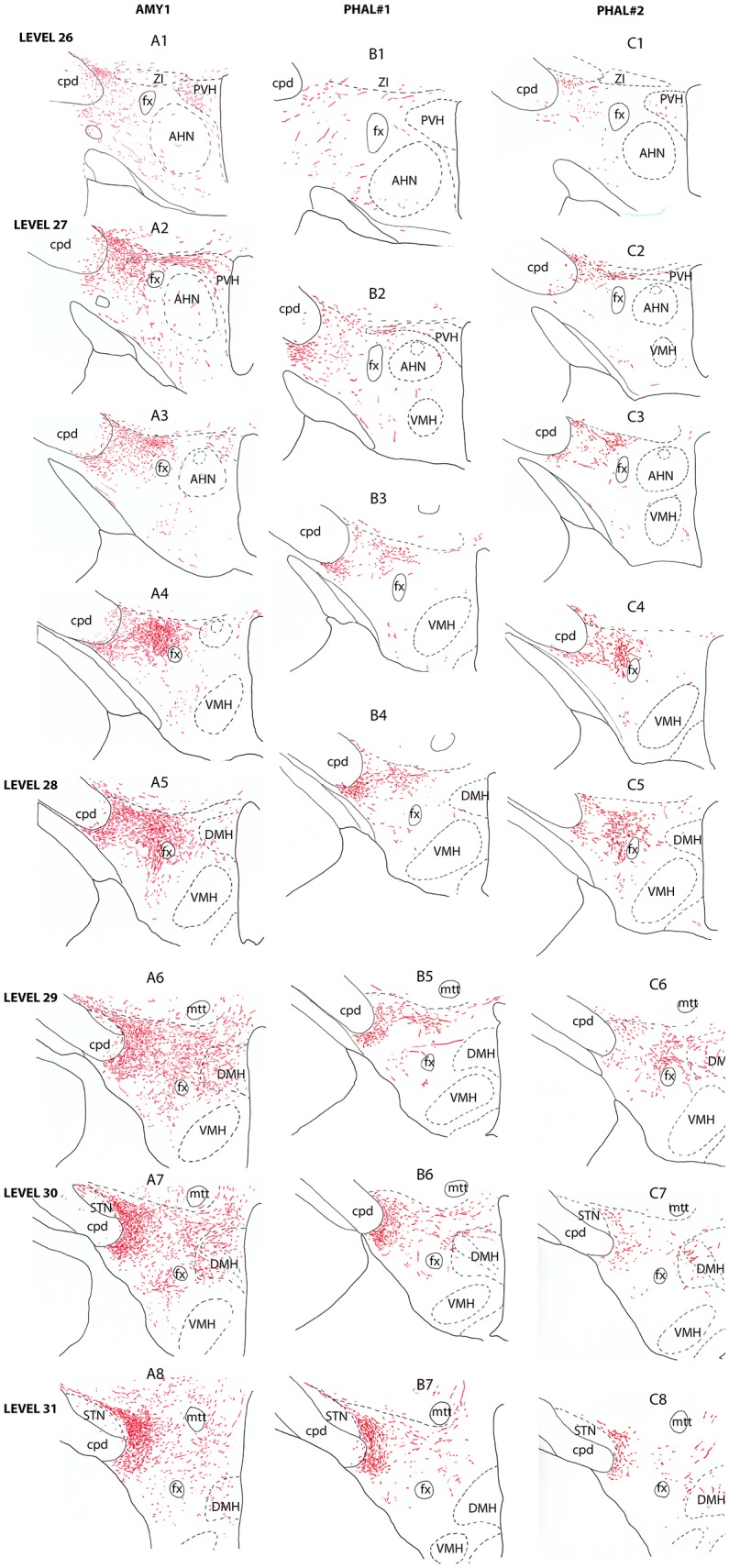
Line drawings describing the distribution patterns of PHAL axons in the LHA of sections corresponding to the levels 26–31 of the Swanson's Brain Maps (28) in experiments AMY1, PHAL#1, and PHAL#2.

Experiment AMY1: As the experiment AMY1 involved most of the CEAm, it labeled the most complete pattern of projections in the LHA. The description of these patterns is illustrated in the Figure 2.

Anterior region of the LHA, e.g., corresponding to the level 26 of the Brain Maps (28), was little innervated (Figure 2A1). A few axons were observed in the paraventricular hypothalamic nucleus (PVH). These axons entered the nucleus by its perifornical part at level 27 (Figure 2A2). The perifornical part of the nucleus is well innervated as well as caudal parvicellular neurons. The LHA contains only a loose innervation at these rostral levels, and most axons resembled to passing axons in the two dorsal and ventral pathways that were described in the work of Barbier et al. (22).

Caudal to the PVH, corresponding to inter-level 27–28 (Figures 2A3–A4, 3B), cells dorsal to the fornix received an intense innervation. Immediately caudal to the PVH (Figure 2A3), this region was quite restricted in size. It concerned cells in the dorsal perifornical LHA. In the next caudal sections, the innervation encompassed the whole dorsal perifornical region as well as a band of tissue immediately lateral to the fornix (Figure 2A5). The innervated perifornical area was largest on sections corresponding to the level 28 of the Brain Maps. Within this region, axons displayed the typical complex aspect with many buttons suggesting that they make many synaptic contacts. We also noted clear pericellular nets around neuronal soma.

**Figure 3:**
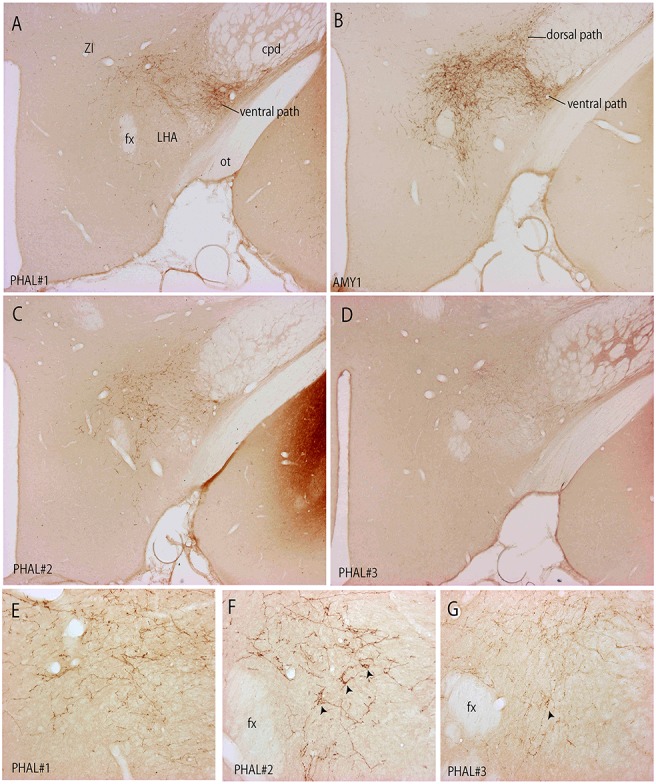
Photomicrographs illustrating the distribution of PHAL axons in the rostral tuberal LHA of the four experiments PHAL#1, AMY1, PHAL#2, and PHAL#4. **(A–D)** Low magnification pictures. Note in A (PHAL#1) that only the ventral pathway is labeled, but axons innervate dorsal LHA regions. By comparison, the dorsal and ventral pathways are clear in the experiment AMY1. Injections in experiments PHAL#2 and #3 resulted in smaller sites and the number of axons is lower than in the two other cases. In the latter **(D)** axons are also thinner and more difficult to see at this magnification. **(E–G)** Higher magnification pictures to illustrate aspects of axons in the experiments PHAL#1, #2, and #3. Note the clear pericellular nets (arrowheads) in the lateral perifornical region of experiment PHAL#2, and even one in PHAL#3. Such figures could not be seen in the experiment PHAL#1. In this last experiment, axons displayed less collaterals and buttons. Therefore, even if the axons were very well labeled, this was interpreted as a weaker innervation of the LHA that than in the other experiments.

In contrary to the perifornical region in which the intensity of the innervation decrease with more caudal tuberal levels, far lateral hypothalamic regions received a more intense innervation by PHAL axons with more caudal hypothalamic levels. Far lateral regions of the LHA contain the ventral and dorsal pathways taken by axons from the CEAm that were described in a previous study (22). At rostral levels (levels 26–28), axons provide a sparse innervation of the ventrolateral and dorsolateral LHA. From levels 29/30, the dorsolateral LHA receives a clear innervation as the morphology of PHAL axons change, displaying a complex organization and many buttons. At the level 30, this innervation is intense in an area adjacent to the subthalamic nucleus (STN) (Figures 2A6–A8).

**Figure 4:**
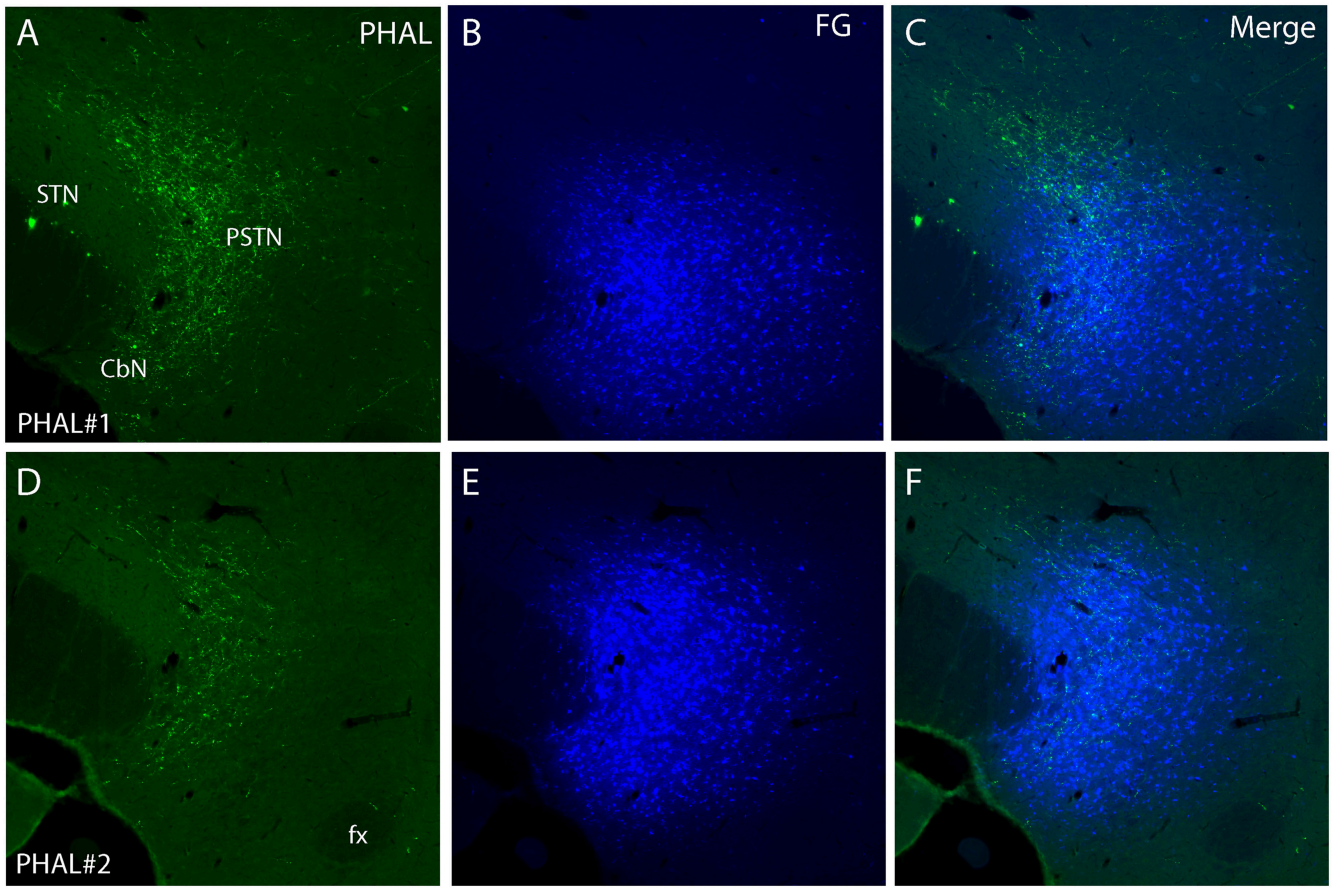
Photomicrographs of PHAL **(A, D)** and FG **(B, E)** dual labeling in fluorescence in experiments PHAL#1 and PHAL#2. Note the innervation of the PSTN region, adjacent to the STN, by PHAL axons. The innervation in experiment PHAL#1 is however more intense than in experiment PHAL#2. A FG injection was aimed at the PSTN in both experiments that slightly involved adjacent STN and LHA areas. (**C, F** Merge images from **A, B** and **D, E** respectively).

Experiment PHAL#1: Although restricted to the cytoarchitectonic borders of the CEAm, the injection site in this experiment was confined to the rostral part of the subnucleus, caudal most perikarya being observed in sections corresponding to the level 26 (Figure 1). Compared to the experiment AMY1, axons essentially run through the ventral pathway (Figures 2, 3A). The innervation of the anterior perifornical region was weaker than that in the preceding experiment and seemed slightly more dorsal (Figures 2B3, B4, 3E). In the caudal LHA, the far lateral area adjacent to the STN was intensely innervated as in experiment AMY1 (Figures 2B6, B7), but again the PSTN appeared as the main target of the projections from this experiment (Figure 4A).

Experiment PHAL#2: The pattern of projections from this injection site centered into the CEAi was very different to that from experiment PHAL#1. Projections coursed through the dorsal and ventral pathways and entered the posterior PVH through its periforniceal part (Figure 2C2). Immediately caudal to this level, an innervation of dorsal perifornical regions was apparent, similar to the experiment AMY1 (Figures 2C3, C4). At inter-level 27–28, the area innervated by these axons clearly involved lateral perifornical regions in addition to dorsal region. Axons formed clear pericellular nets on scattered neurons of this region (Figures 3C,F). At level 29, the perifornical region is less innervated, but axons took a medial direction and a light innervation of the dorsomedial hypothalamic nucleus (DMH) was evident (Figure 2C6). At the levels 30–31, far lateral regions that were intensely innervated in experiment AMY1 and in experiment PHAL#1, were only slightly innervated by axons in experiment PHAL#2 (Figures 2C7, C8). The PSTN was also slightly labeled compared to that in experiments PHAL#1 or AMY1 (Figure 4D).

Experiment PHAL#3: Projections from this experiment are little illustrated, but their distribution patterns were identical to that described for experiment AMY1, although they were less abundant and thinner as the injection site was smaller (Figures 3D,G).

### Co-distribution of PHAL with MCH, Hcrt, and FG

To better characterize the LHA regions innervated in each experiment, series of sections were co-labeled for MCH and Hcrt. Furthermore, in experiments PHAL#1 and PHAL#2, PHAL injections in the CEA were combined with FG injections in the PSTN (Figure 4). Therefore, in these two experiments, PHAL, MCH, and Hcrt immunolabeling were also compared with the distribution of FG retrogradely labeled neurons.

**Figure 5:**
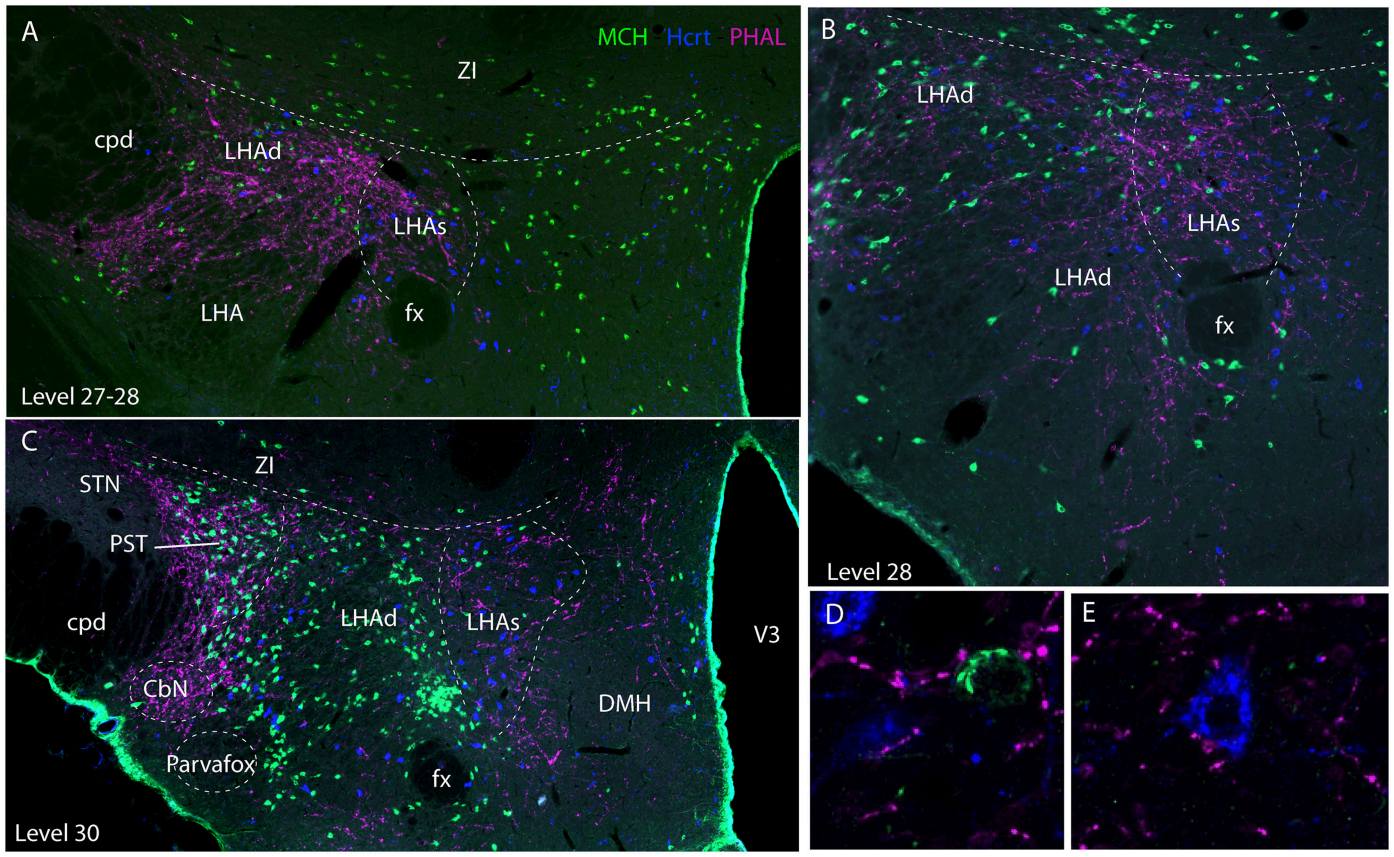
Photomicrographs illustrating the co-distribution of PHAL axons, MCH and Hcrt cell bodies using a triple immunofluorescence procedure. **(A–C)** are low magnification pictures on sections at levels 27 (in fact better seen as inter-level 27/28), 28 and 30. Note the innervation of regions containing MCH and Hcrt cell bodies and corresponding to the dorsal and suprafornical regions of the LHA. However, the ZI, or the caudal perifornical regions (level 30) that are rich in MCH cell bodies but poor in Hcrt neurons are not innervated. By contrast at level 30, the PST that contains a dense group of MCH neurons is intensely innervated. **(D,E)** Higher magnification illustrations showing that buttons by PHAL axons could be seen close to MCH and Hcrt cell bodies. However, it seemed to be mostly *en passant* putative contacts.

Experiment AMY1: Axons followed in the dorsal perifornical region a distribution that by in large corresponded to the distribution of Hcrt cell bodies from levels 28 to 30 (Figure 5). At the level 30, Hcrt perikarya occupy a more medial position compared to the fornix, and PHAL axons in this experiment followed the same pattern, explaining the decrease of innervation of the dorsal perifornical area described in the preceding paragraph. This pattern of distribution of Hcrt cell bodies, corresponded at least partly to the suprafornical part of the LHA described by Hahn (30). The PHAL labeling may be interpreted as an innervation of this part in this experiment. More laterally, axons provided an intense innervation of a triangular shaped region dorsally adjacent to the cerebral peduncle from the levels 30–31. This region corresponded to a condensation of MCH cell bodies and is named PST in the Swanson's Brain Maps (28), see as well Swanson et al. (7) and Hahn (30). However, a dense MCH condensation around the fornix at the same levels was not innervated as well as the MCH rich region in the ZI and sub-incertal territories. At high magnifications, we could see that many MCH or Hcrt cell bodies in the LHAd, s, as well as in the PST, could receive an “en passant” innervation (Figures 5D–E). Pericellular nets provided by axons in the perifornical regions were never observed around MCH or Hcrt neurons.

**Figure 6:**
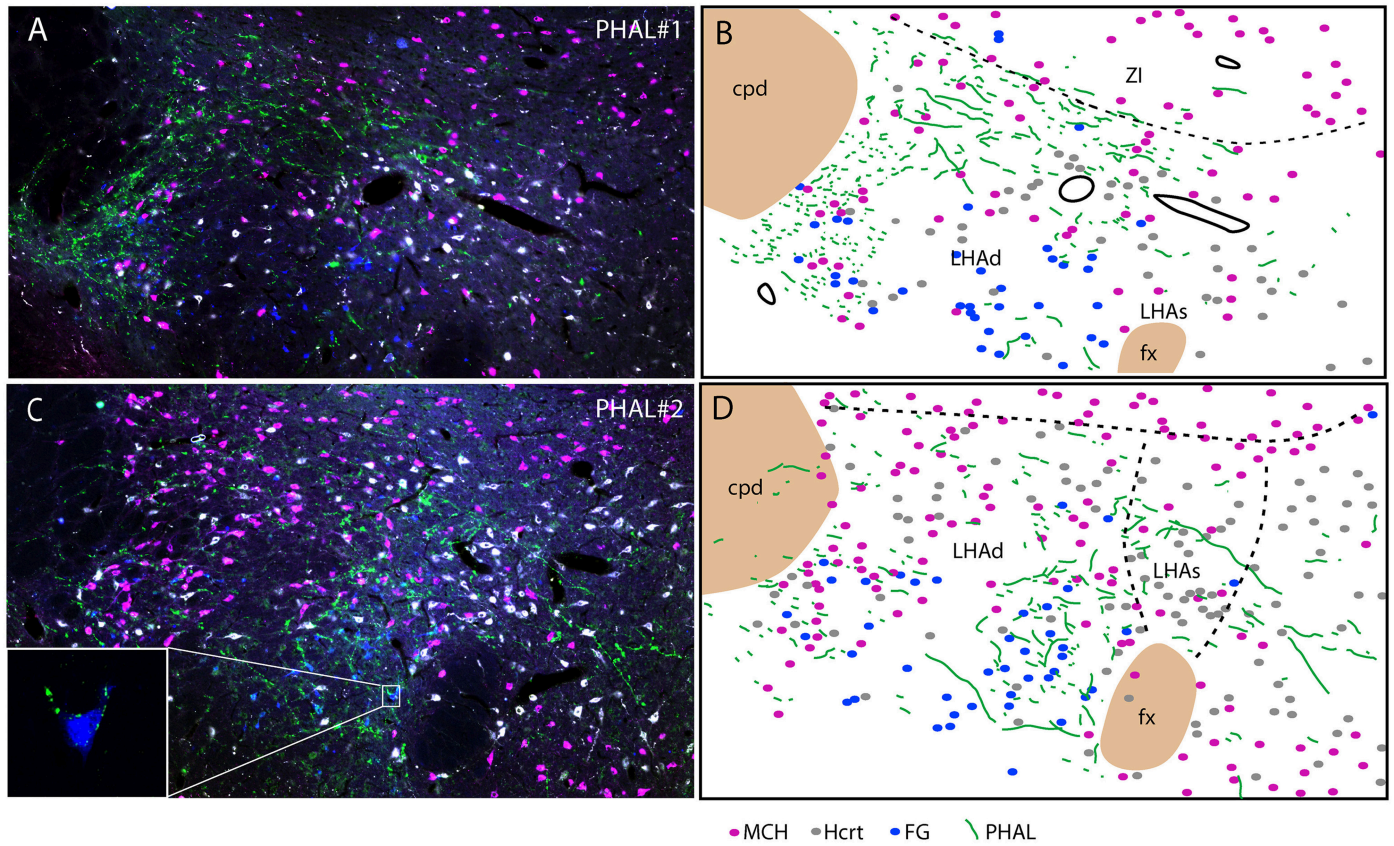
Photomicrographs after quadruple labeling experiments with the detection using immunofluorescence of PHAL, MCH, and Hcrt, the fourth signal being the native fluorescence of the FG. Because it is difficult to separate signals from each other on the picture, we added line drawings that schematize the distribution of these signals. Sections are from experiments PHAL#1 **(A,B)** or PHAL#2 **(C,D)** respectively, and pass approximately through the level 28. Note that the PHAL distribution is distinct in these two cases, with an innervation restricted to the LHAd in experiment PHAL#1, while the LHAs is innervated in the experiment PHAL#2. Furthermore in this experiment, innervation of FG retrogradely labeled neurons from the PSTN are observed. In fact in a few occasion we could see PHAL axons forming pericellular nets around FG-containing cell bodies lateral to the fornix [framed picture in **(C)**, optical slice taken using the Apotome feature of the microscope]. FG labeled neurons were observed lateral to the fornix, in an area that belong to the LHAd, and in the far ventrolateral LHA. However, we cannot rule out the possibility that the labeling of these far latero-ventral neurons could not be the result of an uptake of the FG through dendrites extending into the injection site. In the two experiments, as in the experiment AMY1, only passing axons seemed to contact MCH or Hcrt cell bodies.

Experiment PHAL#1: Projections in this experiment were dorsal in the rostral tuberal LHA and involved an MCH rich zone immediately ventral to the ZI (Figures 6A,B). This zone corresponds to parts of the dorsal region of the LHA in Swanson's Brain Maps. In this experiment, FG was injected in the PSTN. FG retrogradely labeled cells were mostly observed in a ventrolateral corner of the LHA and lateral to the fornix. We observed very few double labeled FG/MCH or FG/Hcrt perikarya. If axons of the ventral pathway could innervate “en passant” ventrolateral FG cells, little correspondence with FG containing neurons lateral to the fornix could be observed.

Experiment PHAL#2: The FG distribution was identical in this experiment to that in the case PHAL#1. PHAL innervation of the suprafornical part was observed, extending into the DMH. Obviously, PHAL axons were more abundant lateral to the fornix where FG retrogradely labeled neurons were observed (Figures 6C, D). Many FG cells appeared targeted by PHAL axons and, in a few instances, we also observed that pericellular nets by PHAL axons surrounded FG retrogradely labeled cell bodies (Figure 6C). Again, in this experiment, a total of < 10 FG cells contained MCH or Hcrt.

## Discussion

The present work illustrates the complex patterns of innervation of the LHA by the CEA. PHAL injections in the rat CEAm and CEAi provided a distinct pattern of innervation of the tuberal region of the LHA. Several comments can be drawn from this observation and will be discussed in the following paragraphs.

### Convergence of projections into the caudal lateral LHA

The caudal lateral LHA, in which we include the PST and the PSTN, receives projections from every PHAL sites in the CEAm and CEAi. These projections are especially intense from the CEAm. This observation confirms results presented in a previous article in which we already mentioned that the PSTN is the main hypothalamic target for the CEAm projections (22). However, the PSTN is part of the premammillary hypothalamus and is devoid of MCH and Hcrt neurons (7, 21, 30). It is also very poor in GABAergic neurons (21). The region just rostral to this nucleus, named PST by Swanson, receives an intense CEAm input as well. This part is rich in MCH cell bodies and therefore GAD containing cells. It is associated to the tuberal LHA that is rich in such MCH and GAD neurons, even if MCH cells may not systematically use GABA as a neurotransmitter (31). Projections into the PST are however less intense than those observed in the PSTN. Finally, the CEAi appears to innervate only moderately both cell groups compared to the CEAm.

### Divergence of projections from the CEAm and CEAi into the LHAd and LHAs

Rostral parts of the LHA received projections from the CEA, but these projections showed different patterns depending on their origin. They essentially concerned areas named LHAd and LHAs by Swanson (28). The LHAs is a bit easier to distinguish as it contains many Hcrt perikarya, few MCH cell bodies and lies dorsal to the fornix at levels 28/29 and then extends dorso-medially to this tract at the level 30. The LHAd corresponds to a larger and latero-dorsal LHA region. Our results suggest that this last region is not homogeneous. FG injections into the PSTN labeled cells in the lateral perifornical region in a rostral tuberal hypothalamus attached to the LHAd in Swanson (28). This region contains few MCH or Hcrt cell bodies and it is obviously innervated by the caudal CEAm and CEAi but not by the rostral CEAm. It is therefore very likely that future chemoarchitectonic and hodologic studies will further subdivide the LHAd.

**Figure 7:**
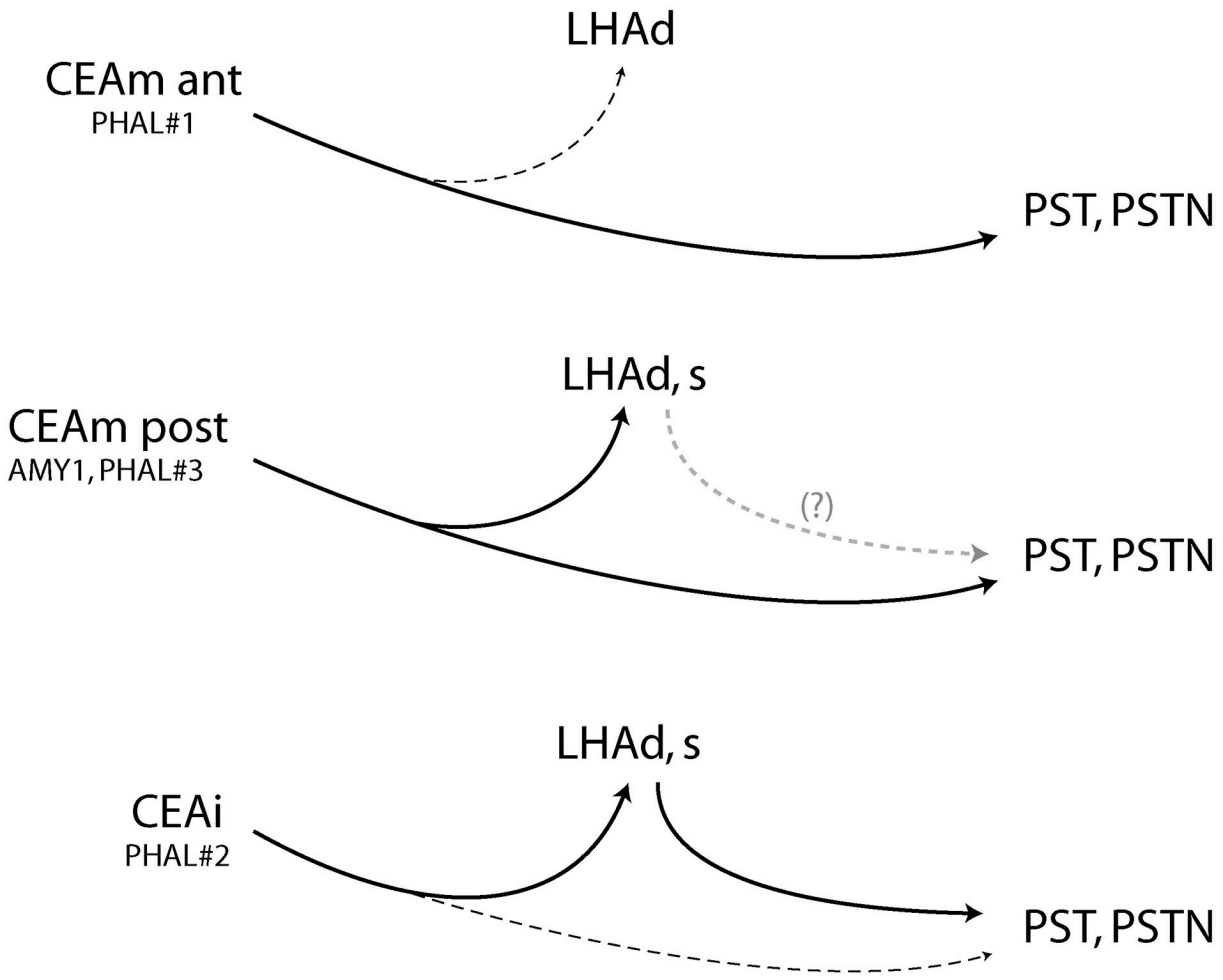
Summary diagram and showing that the CEAm and CEAi send convergent projections into the posterior and lateral LHA but divergent projections into the LHAd, s. After CEAli injection, projections into the PSTN may require a relay into the perfornical LHA, suggesting an intra-LHA network under the control of the CEAi. It may also exist from the caudal CEAm but was not investigated in this study. (Dashed lines represent weak inputs).

Injection in the anterior CEAm (experiment PHAL#1) labeled fewer projections in the LHAd than other experiments and did not innervated the LHAs. Projections from this injection site labeled mainly axons passing through the ventral pathway described by Barbier et al. (22). This pattern is reminiscent of the projections from the CEAm in the study of Gonzales and Chesselet (32) and Bourgeais et al. (33) in which it was shown that such projections traveled in the ventrolateral LHA and provided light LHA innervation. We confirm here that a rostral PHAL injection site in the CEAm is at the origin of such a light pattern of inputs into the anterior tuberal LHA, and this input innervate mostly dorsal aspects of the LHAd. Therefore, as illustrated in the Figure 7, the rostral CEAm innervate lightly the LHAd, does not innervate the LHAs, but sends a heavy input into the PST and PSTN.

Injection sites in experiments AMY1 and PHAL#3 involved more caudal CEAm regions (AMY1 involve both rostral and caudal CEAm). In these two experiments, dorsal and ventral pathways were observed, as described in Barbier et al. (22). Projections, not seen in experiment PHAL#1, extended into the LHAs. Therefore, the caudal CEAm appears to send a heavier input into the rostral LHA than the rostral region of this subnucleus, and this input involved LHAd and LHAs.

The pattern of projections in the LHAd and s in experiment PHAL#2 was reminiscent to that in experiment AMY1 and PHAL#3. We also showed that axons in a region immediately lateral to the fornix and belonging to the LHAd, contained FG retrogradely labeled cells after FG PSTN injection. PHAL axons in experiment PHAL#2 clearly innervated some of these cells, while in a comparable FG injection in experiment PHAL#1, lateral perifornical cells were observed but PHAL axons were not observed close to them. The innervation of such cells in experiment PHAL#2 is interesting as it suggests that the CEAi may indirectly influence the PSTN through an intra-LHA circuit, which can be correlated to the observation of a lighter direct innervation of the PSTN by PHAL axons (Figure 7).

These patterns of projections are supported by literature data. In a recent study about the characterization of the perifornical projections from the CEA (27), we noted that retrograde tracer injections into the caudal lateral hypothalamus labeled many more neurons in the CEAm than in the CEAi, while rostral perifornical injections labeled a dense cluster in the CEAi. The study of Han and Swanson (34) is also interesting as these authors injected retrograde and anterograde tracers into the LHAs. They illustrated that the rostral CEAm contained no retrogradely labeled cells, while the rostral CEAl [corresponding to the CEAi, see (27)], and adjacent caudal CEAm contained such retogradely labeled cells. The paper of Reppucci and Petrovich (35) also noted intense labeling in the rostral CEAl after retrograde tracer injections into the perifornical region.

### Functional considerations

The experimental evidence exposed in this work point to a complex pattern of projection from the CEAm,i into the LHA, and the question of the functional meaning of this observation therefore arises, even if it is very difficult to provide a satisfactory answer at this stage.

The PSTN receives a convergent input from all parts of the CEAm and the CEAi. This nucleus is suspected to play a role in the feeding response. The expression of c-Fos increases in this nucleus when the animal ingests palatable food (21), and other studies suggest it might be part of a satiety circuit (36).

The LHAs has also been little investigated by itself, but its projections were well described in the work of Han and Swanson (34). Several features are reminiscent of those from the CEAm,i. In particular projections into the caudal PVH are similar. This region is also connected to the parastrial nucleus and to the DMH which are also targeted by the CEAm and/or CEAi [see (27)]. Interestingly, this region does not project into the PSTN, and our retrograde injections also failed to label perikarya in this part of the LHA. Han and Swanson extensively discussed the connections of this zone and pointed its association with ingestive behavior (30, 34). As a perifornical structure, there is also a considerable amount of work associating this general region to ingestive behaviors (37–43). In particular, the LHAs contains many Hcrt neurons that seemed targeted by PHAL axons. Hcrt is involved in reward-related food intake and flavor/taste learning through a network that involves the accumbens, the ventral pallidum, the VTA and the amygdala including the CEA (44–49). Our results are in range with these previous findings, but suggests that the caudal CEAm might more directly intervene in the innervation of Hcrt cell bodies in the LHAs.

The LHAd contains a mix of loosely organized MCH and Hcrt cells, but the area lateral to the fornix contains fewer such cells. It is however quite intensely labeled by FG after injection in the PSTN. This area contains neurons expressing neurotensin (50, 51). Neurotensinergic cells are involved in the homeostatic control of food intake and express c-Fos after administration of leptin.

Therefore, the divergent innervation of the tuberal LHA by CEA axons reflects that they innervate cell populations controlling distinct aspects of the feeding response. This hypothesis is speculative, but coherent with the actual anatomical data.

Finally, the PST is filled with MCH cell bodies, and some of our previous works pointed that many spinally projecting MCH neurons are found in this region (8) and may influence autonomic output.

Nonetheless, the distribution of MCH and Hcrt cells being so stereotyped, they prove to be excellent tools to identify and characterize divisions in the LHA, as was stated by Swanson et al. (7) and Hahn (30). Further analysis of the co-distribution of specific afferences with that of these neurons appears to be a good way to better understand a certain parceling of the LHA which may help to understand its organization.

## Author contributions

P-YR conceived and designed the experiments. MB and P-YR performed the experiments and illustration production. MB animal handling. MB, DF, and P-YR analyzed the data and wrote the text.

### Conflict of interest statement

The authors declare that the research was conducted in the absence of any commercial or financial relationships that could be construed as a potential conflict of interest.

## Abbreviations

AAA: Anterior amygdaloid area
ac: Anterior commissure
amc: Amygdalar capsule
AHN: Anterior hypothalamic nucleus
BLAa: Basolateral amygdalar nucleus, anterior part
BLAp: Basolateral amygdalar nucleus, posterior part
BMAa: Basomedial amygdalar nucleus, anterior part
BMAp: Basomedial amygdalar nucleus, posterior part
CbN: Calbindin nucleus
CEA: Central amygdalar nucleus
CEAc: Central amygdalar nucleus, capsular part
CEAl: Central amygdalar nucleus, lateral part
CEAi: Central amygdalar nucleus, intermediate part
CEAm: Central amygdalar nucleus, medial part
CLA: Claustrum
COAa: Cortical amygdalar nucleus, anterior part
cpd: Cerebral peduncle
DMH: Dorsomedial hypothalamic nucleus
ec: External capsule'
EPd: Endopiriform nucleus, dorsal part
EPv: Endopiriform nucleus, ventral part
FG: Fluorogold
fx: Columns of the fornix
GABA: Gamma-aminobutyric acid
GPe: Globus pallidus, external segment
Hcrt: Hypocretin/orexin
IA: Intercaleted amygdalar nuclei
LA: Lateral amygdalar nucleus
LHA: Lateral hypothalamic area
LHAav: Lateral hypothalamic area, anterior region, ventral zone
LHAd: Lateral hypothalamic area, dorsal region
LHAs: Lateral hypothalamic area, suprafornical region
lot: Lateral olfactory tract
MA: Magnocellular preoptic nucleus
MCH: Melanin-concentrating hormone
MEAad: Medial amygdalar nucleus, anterodorsal part
MEAav: Medial amygdalar nucleus, anteroventral part
MEApd-a,b,c: Medial amygdalar nucleus, posterodorsal part sublayers a-c
MEApv: Medial amygdalar nucleus, posteroventral part
mtt: Mammillothalamic tract
NLOT: Nucleus of the lateral olfactory tract
NLOT2: Nucleus of the lateral olfactory tract, pyramidal layer
opt: Optic tract
PHAL: *Phaseolus vulgaris* leucoagglutinin
PVH: Paraventricular hypothalamic nucleus
PST: Preparasubthalamic nucleus
PSTN: Parasubthalamic nucleus
SI: Substantia innominate
sm: Stria medullaris
SO: Supraoptic nucleus, proper
st: Stria terminalis
STN: Subthalamic nucleus
sup: Supraoptic commissures
V3: Third ventricle
vlt: Ventrolateral hypothalamic tract
VMH: Ventromedial hypothalamic nucleus
ZI: Zona incerta.
